# Effects of Different Resuscitation Fluids on Pulmonary Expression of Aquaporin1 and Aquaporin5 in a Rat Model of Uncontrolled Hemorrhagic Shock and Infection

**DOI:** 10.1371/journal.pone.0064390

**Published:** 2013-05-31

**Authors:** Ju Gao, Luojing Zhou, Yali Ge, Shunyan Lin, Jin Du

**Affiliations:** 1 Department of Anesthesiology, Subei People’s Hospital (Clinical Medical College of Yangzhou University), Yangzhou, Jiangsu Province, People’s Republic of China; 2 Department of Scientific Research, Subei People’s Hospital (Clinical Medical College of Yangzhou University), Yangzhou, Jiangsu Province, People’s Republic of China; University of North Dakota, United States of America

## Abstract

**Background:**

To investigate the effects of fluids resuscitation on pulmonary expression of aquaporin1 and aquaporin5 in a rat model of uncontrolled hemorrhagic shock and infection.

**Methods:**

Sixty Sprague-Dawley rats were randomly assigned to five groups, sham operation group (Group C) and four treated groups: no fluid resuscitation group (Group NF), groups resuscitated with Lactated Ringer's (LR),7.5% NaCl (HTS) and Hydroxyl ethyl starch (HES) respectively. Three-phased uncontrolled hemorrhagic shock and infection model was used. Phase I: Massive hemorrhage with a mean arterial pressure of 35–40 mmHg for 60 min, and followed by infection of lipopolysaccharide. Then some animals were resuscitated with solutions mentioned above, until 90 min. Phase II: At hemorrhagic shock 90 minutes, phase II of 60 minutes began with hemostasis and returning of all the initial shed blood. Phase III: Observation phase for 3.5 hours. After phase III, arterial blood gas analysis and the survival rates of the rats were recorded, Wet-to-dry lung weight ratio, BALF protein, pulmonary permeability index, and expressions of aquaporin1 and aquaporin5 were tested.

**Results:**

The expressions of aquaporin1 and aquaporin5 were decreased in treatment groups comparing with sham operation group. Group HES and Group HTS decreased pulmonary vascular permeability and Wet-to-dry lung weight ratio, improved arterial blood gas analysis and survival rates, and attenuated the decreased pulmonary expression of aquaporin1 and aquaporin5 after the “two-hit”, comparing with groups NF and LR,but these beneficial effects were blunted in group HTS.

**Conclusion:**

The expression of aquaporin1 and aquaporin5 may play important roles in formation of pulmonary edema. Resuscitation with HTS and HES, especially HES can reduce lung injury after hemorrhagic shock, partly by up-regulating the expressions of aquaporin1 and aquaporin5.

## Introduction

Traumatic injury, often accompanied by hemorrhagic shock (HS), continues to be the most common cause of death for young people, as well as a significant source of morbidity and mortality for all ages [1], [2].

Infusions of crystalloid and colloid solutions are often the mainstay for the prehospital and inhospital treatments of severe HS. However, it is still a controversy that which would be the better choice for fluid resuscitation after severe HS. The ideal resuscitation fluid would restore the intravascular volume rapidly, reverse the tissue injury effectively and has a beneficial efficacy on mortality [3]. Thus, intensive experiment works are needed to find the more appropriate fluid for resuscitation.The acute lung injury (ALI) or the acute respiratory distress syndrome (ARDS) are the major causes of morbidity and mortality following hemorrhagic shock [4]. Pulmonary edema resulting from injury or infection is one of the pathological conditions of the lung, which is characterized by disrupted fluid transport [5]. However, the exact mechanism responsible for the development of ALI or ARDS remains unclear. Several lines of recent evidence suggest that aquaporins (AQPs) may play a critical role in the pathophysiology of ALI/ARDS. The AQPs are a family of water-selective channels that function to increase plasma membrane water permeability and provide a route for rapid fluid movement [6]. Several known AQPs are expressed in the lung, such as AQP1 and AQP5. AQP1 is expressed in the capillary endothelium and AQP5 is expressed at the apical membrane of alveolar epithelial cells [7]. The observed decrease in permeability in AQP1-knockout mice support a role for AQP1 in the pathophysiology of the lung [8], [9]. In addition, recent studies demonstrated that the expression levels of AQP1 and AQP5 in the lung decreased in viral infection model of pulmonary edema [10], [11]. However, to date, no data is available on the expression of AQPs in the lung in a two-hit model of uncontrolled hemorrhagic shock and infection in rats.Therefore, the purposes of this experiment were (1) to investigate the expression of AQPs in the two-hit model of uncontrolled hemorrhagic shock and infection in rats and (2) to use this model to compare the effects of different fluids resuscitation on ALI and pulmonary expression of AQPs.

## Materials and Methods

This study was approved by Animal Care and Use committee of the School of Medicine, Yangzhou University and was in accordance with the National Guidelines for Animal Care and Use. In the experiment, except for some animals that intolerance to the “two-hit”, and died before the end of the experiment, the other animals all died under deep anesthesia at the end of the experiment by overdose of sodium pentobarbital (150 mg kg^−1^, IP). All animals were ultimately killed by excessive anesthetics because we needed to collect the biological samples from the lung tissue of the animals.

### Animals and Model

60 Sprague-Dawley rats, weighing 250–290 g, were anesthetized with an intraperitoneal injection of pentobarbital sodium (30 mg/kg). The animals were randomly divided into five groups (n = 12 per group), to receive following treatments: (1) sham operation group (group C), only received cannulation and observation; (2) no fluid resuscitation in the “prehospital phase” group (group NF); (3) lactated Ringer’s solution group (group LR), resuscitated with lactate Ringer’s solution in a 3∶1 ratio to the shed blood volume during the “prehospital phase”; (4) 7.5% hypertonic saline group (group HTS), resuscitated with a bolus dose of 4 ml/kg [12] of 7.5% hypertonic saline during the “prehospital phase”; (5)Hydroxylethyl starch group (group HES), resuscitated with Hydroxylethyl starch (Voluven, mean molecular weight 130 kDa; degree of substitution, 0.4, Fresenius Kabi) in a volume equal to the shed blood volume during the “prehospital phase”.All the resuscitation fluids were given immediately after the tail cut.

### Experimental Procedure

A right carotid arterial catheter was placed in rats for continuous hemodynamic monitoring and blood sampling. The mean arterial pressure (MAP) was recorded at baseline (T0), 60 min in prehospital phase (T1), 80 min in prehospital phase (T2), 90 min in prehospital phase (T3) and 60 min inhospital phase (T4), 1 hour in observation phase (T5), 2 h in observation phase (T6), 3.5 h in observation phase (T7). A right jugular venous catheter was placed for administration of resuscitation fluids.Rats were exposed to a two-hit model of uncontrolled HS and infection of three phases [13]:a HS prehospital phase I of 90 min, a resuscitation phase II of 60 min, and an observation phase III of 3.5 h. In phase I, HS was initiated by withdrawal of arterial blood (2 mL of blood per 100 g of body weight), through the right carotid artery catheter until a MAP of 30 mmHg was reached. MAP was maintained at 30–40 mmHg for 60 min by withdrawing or reinfusing the shed blood (kept at 37°C) as required. The shed blood was collected in a glass syringe with heparin (10 IU/mL) and later reinfused during the resuscitation phase. At 60 min of HS, uncontrolled HS was initiated by amputation of 75% of the tail (measured from tip of tail) and followed by mimicked infection by intratracheal administration of lipopolysaccharide (LPS) (E. Coli O55:B5, sigma Chemical, St Louis, MO, USA) 2 mg/kg. During the uncontrolled HS, blood from the tail was collected, measured, and then discarded. Starting at phase I of 60 min, and until phase I of 90 min, animals received different resuscitation fluids according to group assignments. At 90 min, a phase simulating hospital treatment (phase II) began, which lasted for 60 min. The tail stump bleeding was stopped by tail ligation and the tail wound closed. Simultaneously, resuscitation began with the reinfusion of all the blood initially shed. The Phase III was only an observation period with no additional treatments and it lasted for 3.5 h. Throughout the study, all rats breathed spontaneously and a feedback controlled heating system was used to maintain the temperature at about 37°C. Blood samples for the measurement of arterial blood gases were taken before shock (baseline) and at 3.5 h of the phase III. At the end of phase III, the survival rates were recorded. Subsequently, the thorax was opened by a midline thoracotomy and BALF was collected from the left lung through the trachea. The right lung was removed and frozen with liquid nitrogen at a temperature of –180°C for latter assays of AQP1, AQP5, and wet-to-dry lung weight.

### Arterial Blood Gas Analysis

An arterial blood sample of 0.25 mL was used to measure plasma lactate, pH, PO2, and base excess (BE) by a Radiometer ABL 625 Blood Gas Analyzer (Copenhagen, Denmark).

### Measurement of Wet to Dry Lung Weight and BALF/Plasma Protein Ratios

The lung tissue was excised from the rats and then weighed to obtain lung “wet” weights. The obtained tissues are dried in a 60°C oven with desiccant for 72 hours and reweighed, then the W/D ratio was determined. The total protein contents of BALF and plasma were determined by the Bradford protein assay, thus the BALF to plasma protein ratio was determined. Pulmonary permeability index (PPI) values were expressed with the protein concentration in BALF-to-protein concentration in plasma ratio.

### AQP1 and AQP5 Western Blot Analysis

Lung tissues were homogenized with five volumes of lysis buffer containing 1% (vol/vol) NP-40, 0.5% (wt/vol) sodium deoxycholate, and 0.1% (wt/vol) sodium dodecyl sulfate (SDS) in PBS, to which a protease inhibitor, phenylmethylsulfonyl fluoride, 100 µg/mL, was added. The homogenate was left on ice for 30 min before centrifugation. Protein content was estimated and aliquots of 30 µg of homogenate protein were diluted with sample buffer containing 125 mM Tris, 20% (wt/vol) glycerol, and 4% (wt/vol) SDS and separated on 10% (wt/vol) polyacrylamide gels with 0.1% (vol/vol) SDS. Twenty micrograms of protein from the supernatant of each sample was loaded onto a 10% polyacrylamide gel, and transferred to nitrocellulose membranes by the electrophoresis. The membrane was blocked in 3% TBS (Tris Buffered Saline ) solution for 1 h at room temperature and probed with 1∶400 dilution of rabbit anti-aquaporin1(AQP1) and with 1∶300 dilution of rabbit anti-aquaporin5(AQP5) (Santa Cruz Biotechnology Inc, Santa Cruz, CA) in TBS overnight at 4°C. Then the membrane was washed completely and probed by horseradish peroxidase-conjugated goat anti-rabbit IgG at 37°C for 1 h. Protein bands were quantified using the Kodak Digital Science Image Analysis Software (Eastman Kodak, Rochester, NY).

### Lung Histology

Inflation-fixed lungs were washed in phosphate-buffered saline (PBS) three times and bisected for paraffin embedding. Paraffin-embedded lungs were sectioned at 3 mm and stained with hematoxylin and eosin (H&E) for morphologic analysis.

### Statistical Analyses

The results are expressed as means ± standard deviation (SD). With SPSS 13.0 (SPSS Institute, Chicago, IL, USA), one way analysis of variance (ANOVA) followed by the Bonferroni post hoc test was used to compare mean values from five groups. The comparisons of survival rate among groups were made with Kaplan-Meier methods. Differences were considered significant at values of P<0.05.

## Results

### Variation of Mean Arterial Pressure (MAP)

When compared with group NF, the three resuscitation groups resulted in a significantly greater overall increase in MAP (p<0.05) at T2 and T3 time points. However, the groups HTS and HES could restore MAP for a longer time than group LR. The group HTS could not restore the MAP at 2 hours (T6) and 3.5 hours (T7) in the observation phase, while the group HES can restore the MAP almost throughout the experiment (p<0.05) (Table 1).

**Table 1:** The varieties of mean arterial pressure (MAP) at different time points in the rats (12 animals in each group).

Groups	T0 mmHg	T1 mmHg	T2 mmHg	T3 mmHg	T4 mmHg	T5 mmHg	T6 mmHg	T7 mmHg
**C**	**118±8**	115±8	114±9	114±6	111±4	112±7	110±5	108±5
**NF**	**115±9**	38±5*	42±7*	44±6*	77±18*	78±23*	76±27*	73±25*
LR	116±13	36±5*	54±9* △	69±12* △	83±14*	83±16*	84±12*	81±19*
HTS	115±12	36±4*	62±14* ^△▴^	74±15* △	94±16^△▴^	93±15* △	89±19*	83±10*
HES	117±12	38±3*	63±10* ^△▴^	77±14* △	98±13^△▴^	96±14* ^△▴^	96±13* ^△▴^	93±12* ^△▴^

The groups and the time points defined in the text, Data are means ± standard deviation (SD),

* *P*<0.05, v/s Group C,

△ *P*<0.05 v/s Group NF,

▴ *P*<0.05 v/s Group LR.

### Arterial Blood Gas Analysis

There was no obvious differences in the results of the arterial blood gases analysis at baseline among the five groups (data not show). When compared with group C, four treatment groups resulted in significantly greater abnormity in Lactate, pH, PaO2, HCO3-, and base excess (p<0.05) at the end of the experiment (Table 2). However, the arterial blood gases were not different between groups NF and LR (p>0.05), and were significantly better in group HES, when compared with the groups NF and LR (p<0.05). The arterial blood gases of group HTS were also better than group NF, but there was no difference in the Lactate level and base excess when compared with group LR. The blood lactate concentration followed the sequence: group NF> group LR> group HTS> group HES (Table 2).

**Table 2:** The results of arterial blood gases at the end of the experiment (6 animals in each group).

Groups	Lac(mmol/L)	pH	PaO2(mmHg)	HCO3^−^(mmol/L)	BE
C	1.415±0.274	7.343±0.022	140.50±23.59	22.550±1.44	-2.00±0.63
NF	14.468±3.094△	7.008±0.077△	58.33±12.11△	9.453±1.916△	-21.67±3.01△
LR	12.403±3.322△	7.099±0.081△	67.17±9.20△	13.067±1.045△	-19.17±3.66△
HTS	9.785±1.059^△+^	7.210±0.453^△+▴^	82.17±7.52^△+▴^	16.433±2.438^△+▴^	-14.67±3.07^△+^
HES	8.392±1.306^△+▴^	7.246±0.046^△+▴^	81.33±4.84^△+▴^	17.467±2.356^△+▴^	-10.17±2.47^△+▴^

The groups defined in the text, Data are means ± standard deviation (SD),

△ *P*<0.05 v/s Group C,

+ *P*<0.05 v/s Group NF,

▴ *P*<0.05 v/s Group LR.

### Wet to Dry Lung Weight Ratio, BALF and BALF/Plasma Protein Ratio

The W/D ratio, BALF Pro and PPI were all significantly increased in the four treatment groups (p<0.05), but there was no difference between groups NF and LR (p > 0.05). Groups HTS and HES significantly reduced the W/D ratio and PPI compared with the other two treatment groups (p<0.05). Meanwhile, group HES had the lowest amplitude in W/D ratio, BALF Pro and PPI among the treatment groups (Table 3).

**Table 3:** Measurements of W/D, BALF pro, PPI in the five groups (8 animals in each group).

Groups	W/D (%)	BALF Pro (mg/ml)	PPI (%)
C	4.085±0.090	52.82±9.41	1.285±0.191
NF	4.752±0.119△	150.00±15.27△	4.0817±0.328△
LR	4.668±0.107△	142.25±14.69△	3.8150±0.337△
HTS	4.408±0.092^△+▴^	128.10±12.97^△+^	3.177±0.1554^△+▴^
HES	4.272±0.095^△+▴^ *	118.82±8.91^△+▴^	3.132±0.237^△+▴^

The groups defined in the text, Data are means ± standard deviation (SD).W/D: Wet to dry lung weight ratio; BALF pro: Protein concentration in the BALF; PPI: Pulmonary permeability index, valued as BALF/Plasma protein ratio.

△ *P*<0.05 v/s Group C;

+ *P*<0.05 v/s Group NF;

▴ *P*<0.05 v/s Group LR;

* *P*<0.05, Group HES v/s Group HTS.

### Analysis for AQP1, AQP5 Expressions in Lung Tissues

**Figure 1:**
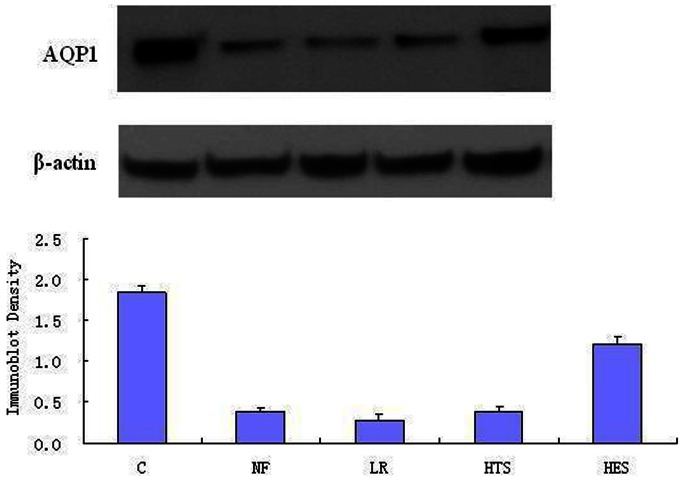
Western Blot Analysis for AQP1 in the lung tissues. Protein were extracted and subjected to Western blot analysis with use of polyclonal antibody against AQP1. Lanes are as follows: 1, Group C; 2, Group NF; 3, Group LR; 4, Group HTS; 5, Group HES group. Data presented are means ± standard deviation (SD) of three or four separate experiments. ^△^ *P*<0.05 versus Group S; ^+^ *P*<0.05 versus Group NF; *P<0.05 versus Group LR; ^▴^ *P*<0.05 versus Group HTS.

**Figure 2:**
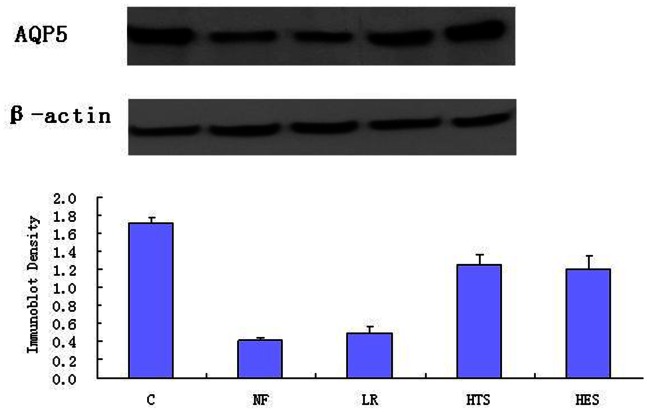
Western Blot Analysis for AQP5 in the lung tissues. Protein were extracted and subjected to Western blot analysis with use of polyclonal antibody against AQP5. Lanes are as follows: 1, Group C; 2, Group NF; 3, Group LR; 4, Group HTS; 5, Group HES. Data presented are means ± standard deviation (SD) of three or four separate experiments. ^△^ *P*<0.05 versus Group C; ^+^ *P*<0.05 versus Group NF; *P<0.05 versus Group LR.

The AQP1 and AQP5 expressions were significantly decreased in four treatment groups in comparison to group C (p<0.05). There were no significant differences in AQP1 and AQP5 expression between groups NF and LR. The groups HES and HTS attenuated the HS-induced decreased pulmonary expression of AQP1 and AQP5 after the “two-hit”. Both the pulmonary expressions of AQP5 and AQP1 was much higher in group HES than groups NF and LR (P<0.05). However, group HTS only showed an obvious increase in AQP5 expression when compared to groups NF and LR (Fig. 1, Fig. 2).

### Histopathological Studies

**Figure 3:**
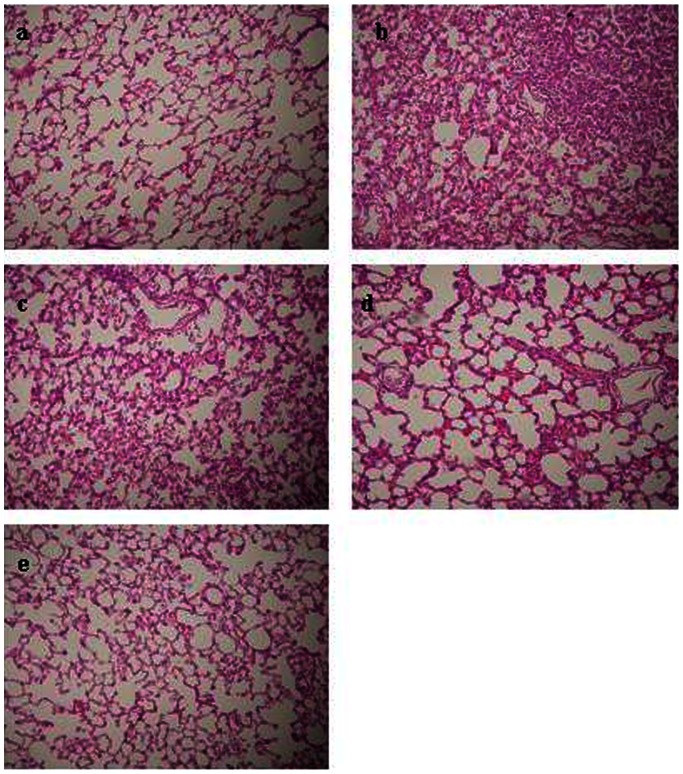
Histology of representative lung sections of rats. Significant numbers of inflammatory cells migrated into the alveolar spaces, the alveolar walls showed destructive changes and alveolar congestion in the NF and LR group (panel b, panel c). The attenuation of lung injury with HTS and HES treatment group was noted (panel d, panel e). Five rats were examined in each group. Sections were stained with hematoxylin and eosin stain. Original magnification: ×100.

The pathological changes of lung were examined with light microscope. No inflammatory change was observed in group C (Fig. 3a), and the lung appeared to remain intact. However, a mass of inflammatory cells infiltrated and migrated into the alveolar spaces and around the vessels, the alveolar wall thickened and showed destructive changes in groups NF and LR (Fig. 3b and 3c). These observations of lung injury seen in the two groups above were obviously attenuated in groups HTS and HES, whichwere manifested by less alveolar septal thickening, less inflammatory cells infiltration, and less alveolar congestion (Fig. 3d and 3e).

### The Survival Rate of Rats after Observation Phase III

At the end, the survival rates of rats were 100%, 60.3%, 75%, 91.7%, and 91.7%, respectively in group C, group NF, group LR, group HTS and group HES. The survival rates of groups HES and HS were significantly enhanced, when compared to groups NF and LR.

## Discussion

ALI is characterized by increased permeability of the alveolar capillary barrier, which allows an influx of protein-rich fluid into the air spaces, causing pulmonary edema [14], [15]. Several lines of evidence indicates that AQPs may be important in physiological and pathophysiological processes in the lung [16]. They provide a major pathway for osmotically driven water movement across epithelial and microvascular barriers in the lung. The fluid movement is significantly reduced, 10-fold by deletion of AQP1 or AQP5 and reduced even more by deletion of AQP1 and AQP5 together [9]. Additionally, in aged rats, the reduced lung water transport rate was associated with the downward regulation of AQP1 and AQP5 [17].Our present study showed that there was significantly decreased expression of AQP1 and AQP5 in the rat lungs after “two-hit” model, which was consistent with the increased W/D ratio, as well as the obvious histological alterations. Previous studies suggested that the decrease of AQP1 may play a compensatory role after the lung injury. Alternatively, a decrease in both AQP1 and AQP5 may contribute to edema by essentially reducing the transcellular rate of removal of excess water, thereby effectively trapping water in the alveolar and interstitial spaces.These changes in AQP expressions either may represent a response to inflammation associated pulmonary edema or may be causal in the formation of pulmonary edema.

HS is a major cause of death in the setting of trauma. Infusion of crystalloid and colloid solutions is often the mainstay for the prehospital and inhospital treatment of severe HS. However, there is still considerable controversy about which fluid is better for restoring intravascular volume rapidly, reversing tissue injury effectively, and having a beneficial efficacy on mortality. Previous studies [18]–[21] supported more evidence of the harmful effects of large-volume Ringer’s lactate solution, including initiation of inflammatory cascade, neutrophil activity, as well as increased mortality. HES130/0.4, as a new colloid solution, has been used extensively. Some researchers demonstrated that HES 130/0.4 significantly attenuated neutrophil recruitment and subsequent ALI by decreasing cytokines and nuclear factor-kB activation [22]. Our present study illustrated that, in a rat model of uncontrolled HS and infection, resuscitation with HES 130/0.4 significantly attenuated the HS-induced decreased expression of AQP1 and AQP5 in the lung tissues. Concomitantly, the W/D ratio and PPI, which are used as indicators of pulmonary microvascular permeability, decreased significantly in group HES. Therefore, it is considered that the protective effects of HES 130/0.4 on endothelial barrier dysfunction and high epithelial permeability after uncontrolled HS and infection, to some extent, in part by upregulating the expression of AQP1 and AQP5.

It is well established that HS triggers the immune system and increases inflammation [23], and neutrophil activation [24]. Thus, it is generally believed that HS contributes to the pathogenesis of ARDS or ALI. In our study, serious ALI was induced by the two-hit model, which was confirmed by the histological observations. When compared to the groups NF and LR, the protective effects of HES and HTS were also evident by arterial blood gas analysis and histologic examinations of the lung, which showed that HS-induced interstitial edema and neutrophil infiltration were further improved by HTS, and especially by HES resuscitations. Thus, the two groups above would have higher survival rates of rats. However, some previous studies have demonstrated that HES may be associated with excess mortality in critically ill patients [25], [26]. It seems to be contradictory to our experimental results. The probable reasons for the differeces has been analysed as follows. (1) New data indicate that mortality rates may be increased after high cumulative doses of HES infusion [27], [28], and in our experiment, HES 130/0.4 was given only in a volume equal to the shed blood. (2) HES safety studies should be large-scale studied in patients at risk with acute need for volume therapy, and with long enough study periods to detect differences in mortality and morbidity. However, the observation time in our study was only 3.5 h, which was not too short. (3) previous clinical studies usually used older HES preparations, such as HES 200/0.5, while in our experiment, HES 130/0.4 was always used as a third -generation resuscitation fluid. Previous study [29] demonstrated that HES 130/0.4 is safer than older HES preparations, because of its different pharmacokinetic properties.

Additionally, upregulated expressions of AQPs were found in groups HTS and HES when compared with groups LR and NF, and were assumed to be one of the possible mechanisms for preventing lung injury in the two-hit model. However, the exact mechanisms responsible for upregulating the expression of AQPs in groups HTS and HES are currently unknown. Hoffert et al also found that when in the hypertonic solutions, the expression of AQP5 promoted in the lung epithelial cells of rat [30]. Previous studies confirmed that the HES 130/0.4 significantly attenuated inflammation and subsequent ALI by decreasing TNF-α and other cytokines activation in a rat sepsis model[31], [32]. The decrease of TNF-α may induce direct promotion of AQPs [33]. Additionally, Towne et al demonstrated that in the lung epithelial cells treated with TNF-α, the expression of AQP5 decreased to 50% of the normal level [34]. Thus, we considered that the decrease of TNF-α treated with HES may induce the upregulation of AQPs.

The experiment also made a comparison with the HES and HTS, and found that HES was more effective than HTS in preventing ALI in the two-hit model. The severity of histological lung damage, pulmonary microvascular permeability (W/D) and plasma lactate, however, was much greater in group HTS than in group HES. There are some possible explanations. First, from our study, the expression of AQP1 was much higher in group HES than group HTS, and the HTS resuscitation could not upregulate the expression of AQP1 in comparison to groups LR and NF. Previous studies demonstrated the reduced lung water transport rate was associated with the downward regulation of AQP1 [9]. Second, of particular note, inflammatory response after the shock is considered to be a key step leading to tissue damage, and resuscitation with HES was more effective in anti inflammation [35].

Our experimental protocol has several limitations. First, the observed period of our study was limited to 3.5 h. It was insufficient to evaluate the long-term effects of different resuscitation fluids on HS-induced ALI. Further studies are required to assess whether HES can attenuate HS-induced ALI in a longer observation time. The other limitation was that the study did not determine the widespread signaling mechanisms, which are responsible for decreasing and regulating the expression of AQPs after the two-hit model. Clearly, intensive experimental efforts are needed to investigate the exact signaling mechanisms above.

### Conclusion

The current experiment showed that in a rat two-hit model of hemorrhagic shock and infection, the decreased expressions of AQP1 and AQP5 may play an important role in acute lung injury. The altered expression was consistent with the severity of pulmonary edema. The fluid resuscitations with hydroxyethyl starch may be the better choice for preventing ALI after severe HS and infection, in part by up-regulating the expressions of AQP1 and AQP5.
